# Sex differences in color preferences transcend extreme differences in culture and ecology

**DOI:** 10.3758/s13423-014-0591-8

**Published:** 2014-02-26

**Authors:** Piotr Sorokowski, Agnieszka Sorokowska, Christoph Witzel

**Affiliations:** 1 University of Wrocław, Dawida 1, 50-527 Wroclaw, Poland; 2 School of Psychology, University of Sussex, Pevensey II, BN1 9QH Brighton, UK

**Keywords:** Biological components, Color perception, Color preference, Cross-cultural comparison, Sex differences

## Abstract

At first glance, color preferences might seem to be the most subjective and context-dependent aspects of color cognition. Yet they are not. The present study compares color preferences of women and men from an industrialized and a remote, nonindustrialized culture. In particular, we investigated preferences in observers from Poland and from the Yali in Papua, respectively. Not surprisingly, we found that color preferences clearly differed between the two communities and also between sexes. However, despite the pronounced cultural differences, the way in which men and women differed from each other was almost the same in both cultures. At the same time, this sexual contrast was not specific to biological components of color vision. Our results reveal a pattern of sexual dimorphism that transcends extreme differences in culture and ecology. They point toward strong cross-cultural constraints beyond the biological predispositions of nature and the cultural particularities of nurture.

**Electronic supplementary material:**

The online version of this article (doi:10.3758/s13423-014-0591-8) contains supplementary material, which is available to authorized users.

## Introduction

Preferences shape choices, and choices orient our behavior. Understanding color preferences gives insight into the role of color in guiding the observers’ interaction with their visual environment. For example, the evolution of color vision plays an important role in the identification of preferable targets of foraging (e.g., Regan et al., 2001). Moreover, red has a special impact on intellectual (e.g., Elliot, Maier, Moller, Friedman, & Meinhardt, 2007) and physical (e.g., Hill & Barton, 2005) performance. Finally, colors may influence consumers’ product preferences and choices (for a review, see Sable & Akcay, 2011). In all these cases, colors affect the beholders' motivations and, hence, shape their behavior. For this reason, insight into color preferences provides a link between color cognition and the beholders’ choices and actions.

But aren’t color preferences just very subjective and personal? Apparently not. First of all, several studies observed an overall proclivity for blue (e.g., Saito, 1996; review in Crozier, 1999). Moreover, some studies found systematic differences between women and men (e.g., Ellis & Ficek, 2001; Guilford & Smith, 1959; Palmer & Schloss, 2010a). However, it seems difficult to establish a simple pattern of sexual differences across studies—in particular, since different studies measured color preferences with different samples of colors.

Now, a recent study showed that color preferences are systematically related to the affective response to objects in the environment (Palmer & Schloss, 2010a, 2010b); observers tend to prefer colors with which they associate more preferable objects. However, this approach does not completely explain the differences between men and women (Taylor & Franklin, 2012).

Another study succeeded in modeling color preferences of English and Chinese observers through the second-stage mechanisms of color vision (Hurlbert & Ling, 2007). The second-stage mechanisms are implemented by the retinal ganglion cells and provide the basis for subsequent processes of human color vision. Since they are determined by physiology rather than by the beholder’s experience and culture, Hurlbert and Ling called them “biological components.” Although color preferences differed between English and Chinese, the way in which color preferences differed between women and men was similar in both groups. The authors concluded that the universal role of these biological components may be the source of cross-cultural regularities of sex-specific color preferences.

However, the two chromatic biological components used by Hurlbert and Ling (2007) represent the complete perceptual color space for their set of equiluminant colors. As a result, any gradual change of preferences across similar colors ranging between the most (maximum) and least (minimum) preferred colors must result in a correlation with the dimensions that represent color similarity, as was the case in that study. The question remains whether sexual differences in color preferences always change gradually across colors and whether the relative importance of each of these axes is a particular feature of sexual differences that is stable across cultures.

Moreover, all these studies have been conducted among industrialized societies. The two aforementioned studies mainly involved Americans and Japanese (Palmer & Schloss, 2010b) and English and Chinese who lived in the U.K. (Hurlbert & Ling, 2007). Other studies have compared different Asian societies (e.g., Saito, 1996). However, all these societies are part of the global communication network that involves intercultural exchange—for example, via the Internet, TV, and tourism. Gender-specific communication networks could even produce cross-cultural sexual patterns in these societies. So, in all these studies, commonalities across cultures may just be due to cultural trends in color preferences that are shared between observers through global communication flows. However, if there are universal determinants of color preferences, cross-cultural regularities should also appear in a remote, traditional, nonindustrialized community, not exposed to global communication.

A recent study has tried to extend the findings for industrialized societies to a nonindustrialized, remote culture (Taylor, Clifford, & Franklin, 2012). Taylor and colleagues studied the Himba in rural Namibia and compared them with British observers. They found that the Himba color preferences contradicted both the Palmer and Schloss model through associated object preferences and Hurlbert and Ling’s biological component model. Instead, chroma (i.e., the amount to which a hue differs from gray) was the main predictor of Himba preferences. In fact, Himba mainly preferred saturated (high-chroma) over unsaturated (low-chroma) colors with little variation of color preferences across hues. In sum, Himba color preferences did not follow any cross-cultural pattern, because they were mainly determined by how “colorful” the colors were.

However, the biggest challenge of such a cross-cultural comparison consists in ensuring that the nonindustrialized observers accomplish the experimental task in a way that is comparable to the industrialized culture’s performance. It is possible that the dominant influence of chroma on Himba color preferences is due to the fact that Taylor and colleagues (2012) presented stimulus colors on a computer screen. For a truly nonindustrialized, remote culture, a computer screen is a very strange object. In particular, while most colors in nature are surface colors (i.e., colors that result from the absorption of light during the reflectance from a surface), the computer screen shows emitted colors (i.e., colors produced by a light source). The opportunity of seeing highly saturated, luminous colors in this way should be fascinating for a beholder who never sees anything similar in everyday life. As a result, the failure of Taylor and colleagues (2012) to reveal cross-cultural regularities could be merely due to the mode of stimulus presentation.

Here, we also compared a nonindustrialized, remote community with a modern, highly industrialized, and globalized society. In particular, we compared color preferences in women and men from the Yali tribe in Papua and from Polish observers. In contrast to Taylor and colleagues (2012), we used surface colors and kept the task as simple as possible so as to make the task more accessible for nonindustrialised observers. We examined whether color preferences differed between the two cultures and how the color preferences differed between men and women in the two cultures. Finally, we tested whether the biological component model may account for sex differences in color preferences in Polish and Papuan observers.

## Method

### Participants

The first group of participants were 108 Yali from Papua (Indonesia). They inhabit Yalimo, the mountainous terrain east of the Baliem valley. The study was conducted in a few small mountain villages. Life in these villages is fundamentally different from the life in an industrialized society. (e.g., they do not have TV, electricity, or running water or any other modern commodities). The Yali can be described as a population with a minor contact with Western culture; due to the remote location of their dwellings and difficult access, very few tourists have visited their region (the only access routes to the Yali territory are via private or chartered aircraft or a several days long trek through the mountains). Even though Christianity is present in this region, Yali have still preserved their traditional lifestyle, including polygyny or clothing (some men wear only traditional koteka covering their penis) (see Sorokowski, Sorokowska, & Danel, 2013, for further details). The second group were 200 observers in the city of Wroclaw, Poland. Poland is a country in Central Europe with a GDP of about $14,000 per capita and is part of the European Union. It is representative for a modern industrialized society.

In each group, one half were women. Yali women were between 25 and 59 years of age (*M* = 38.4, *SD* = 8.7), and men between 19 and 50 years (*M* = 35.6, *SD* = 7.6). The age was self-estimated; the majority of participants did not know exactly how old they were. In Poland, women were between 19 and 55 years of age (*M* = 31.4, *SD* = 9.8), and men between 19 and 56 years (*M* = 34.4, *SD* = 10.0).

### Stimuli and procedure

**Fig. 1:**
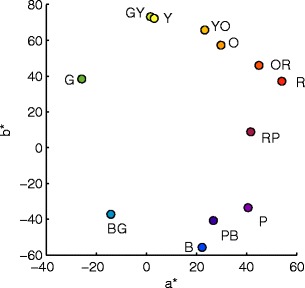
Stimulus colors in CIELAB. Capital letters refer to IDs of stimulus colors: G = green, Y = yellow, O = orange, R = red, P = purple, B = blue; two letters refer to colors in between, such as GY = green-yellow. See Table S1 for exact chromaticity coordinates. Note that colors were chosen to correspond to the prototypes of color names

We used a printed color wheel consisting of six colors that corresponded (approximately) to the prototypes of red, orange, yellow, green, blue, and purple and six colors in between these hues. The exact measured chromaticity coordinates of the stimuli are provided in table S1 of the supplementary material, and the corresponding CIELAB coordinates are illustrated in Fig. 1.

We asked the participants to point first to the color they like most (favorite color) and then to the one they like least (least favorite color). In Yalimo, a Papuan assistant (from the Dani tribe) interviewed the participants in their own local dialects.

## Results

For each of the stimulus colors (*n* = 12), we calculated the relative frequency of observers that chose it as the favorite or least favorite color, respectively. We applied chi-square statistics to test whether the relative frequencies of choices were significantly different from an equal distribution of frequencies across colors and groups. Moreover, we calculated correlations between the relative frequencies of each group. While least favorite color choices were noisier than favorite color choices, they were in line with the main results of the favorite color choices. For this reason, we concentrate on the presentation of the favorite color choices here and provide the additional analyses of the least favorite colors in the “Least Favourite Colours” section of the supplementary material.

### Cultural differences

**Fig. 2:**
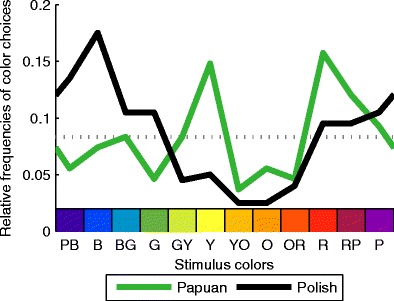
Cultural differences. Relative frequencies of favorite color choices are shown along the *y*-axis. The *x*-axis represents stimulus colors going from purple-blue (PB) to purple (P). For illustration, colors along the *x*-axis correspond to the RGB values of the stimulus colors. Industrialized Polish (black curve) and remote, nonindustrialized Papuan (green curve) observers differ strongly in their color preferences. The dotted gray curve corresponds to chance level. For least favorite choices, see Fig. S2. Polish observers tend to prefer bluish, Papuan observers red and yellow colors

Figure 2 presents the relative frequencies of favorite color choices for the two cultures. Polish observers chose blue most often (17.5 %) and yellow-orange least often (2.5 %) as the favorite color. In contrast, Yali observers most frequently chose red (15.7 %) and yellow (14.8 %) as their favorite color. Like the Polish observers, they chose yellow-orange most rarely as their favorite color (3.7 %).

Polish and Papuan favorite color choices were significantly different, *χ* ^2^(11, *N* = 308) = 27.3, *p* <.01. This evidence for cross-cultural differences is further supported by the absence of a correlation between the Polish and Papuan profile of favorite color choices, *r* = .11, *p* = .74, *n* = 12. At the same time, the cross-cultural differences seem to be systematic in that they were similar for the male and female subgroups: The difference between the relative frequencies of Polish and Papuan favorite color choices correlated highly between men and women, *r* = .86, *p* < .01.

### Sex differences

The color choices of all women and all men are illustrated by Fig. S1 of the supplementary material. Taking both cultures together, women and men differed significantly in the relative frequencies of their favorite color choices, *χ* ^2^(11, *N* = 308) = 50.2, *p* < .001. Women preferred red (21 %), while men preferred blue (19 %). When analyzing the Yali and the Polish groups separately, the difference between men and women was significant in the Yali group, χ^2^(11, *N* = 108) = 21.8, *p* = .03, as well as in the Polish group, *χ* ^2^(11, N = 200) = 31.2, *p* = .001.

**Fig. 3:**
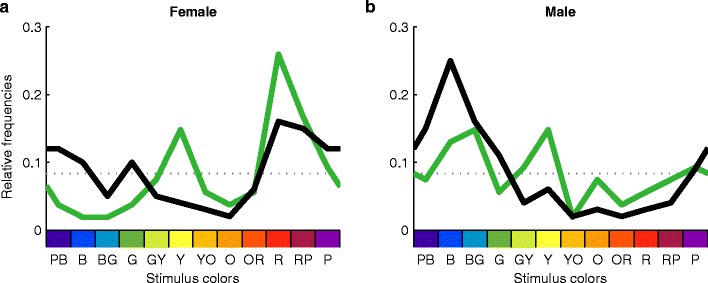
Sex-specific preferences across cultures. Relative frequencies of Papuan (green curves) and Polish (black curves) favorite color choices are shown separately for women (**a**) and men (**b**). Format is as in Fig. 2; least favorite choices in Fig. S3. Within each culture, male and female curves differ significantly, and across cultures, the sex-specific curves in each panel correlate

Figure 3 shows the favorite color choices in each culture separately for women (panel a) and men (panel b). There were commonalities between Polish and Yali women and between Polish and Yali men. The choices of favorite colors correlated significantly between Polish and Papuan men, *r* = .58, *p* = .0496, and almost significantly between Polish and Papuan women, *r* = .54, *p* = .07. Taken together, these observations reflect systematic sex differences and sex-specific patterns across cultures.

**Fig. 4:**
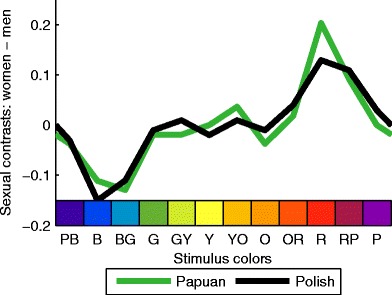
Sexual contrasts. Differences between women’s and men’s relative frequencies of choosing the favorite color are shown along the *y*-axis. For least favorite choices, see Fig. S4. The sexual contrasts are highly correlated between Polish and Papuan observers

The most impressive result, however, is illustrated by Fig. 4. The green curves show the sexual contrasts for Yali, the black curves for Polish observers. Sexual contrasts are the differences between the relative frequencies for men and women. For example, the sexual contrasts for favorite colors of Yali observers correspond to the differences between the black curves in panels a and b of Fig. 3. These sexual contrasts were extremely similar across the two ethnic groups. The correlation between the Polish and Papuan sexual contrasts explained 86 % of the common variance, *r* = .93, *p* < .01.

### Biological components

We modeled the sex differences through the second-stage mechanisms. There are three second-stage mechanisms. The first contrasts the information from long-wavelength and middle-wavelength cones (L–M), which corresponds to a greenish to reddish axis. The second contrasts the activity of short-wavelength cones to the sum of the activity of the other cones [S–(L+M)] and corresponds to a yellowish to bluish axis. The biological component model was originally applied to equiluminant stimuli and, hence, included only these two chromatic dimensions. Since our stimuli varied in luminance, we also tested the impact of the luminance axis (L+M).

We calculated the weights of the measured chromaticity and luminance coordinates of our stimuli (cf. Table S1) along these components. First, we examined the correlations between these weights and the color preferences of our Polish observers in order to compare them with the results Hurlbert and Ling (2007, p. R625) obtained with the English and Chinese observers. The stimulus weights along the L–M component correlated negatively with the favorite color choices, *r* = −.83, *p* < .001. The weights along the S–(L+M) component correlated positively with favorite color choices, *r* = .81, *p* = .001. Like the English and Chinese observers, Polish observers preferred colors at the greenish pole of the L–M component and at the bluish pole of the S–(L+M) component. All three biological components explained 76 % of the variance in Polish favorite color choices, *R* ^2^ = .76, *F*(3, 8) = 8.3, *p* = .01.

**Fig. 5:**
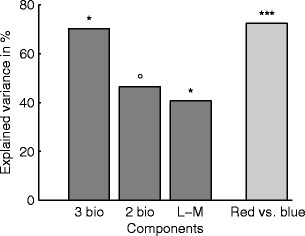
Perceptual components of sexual contrasts. The height of bars represents the variance of overall sexual contrasts (differences between curves in Fig. S1) explained by different models. The “3 bio” model included all three biological components, the “2 bio” model the two chromatic biological components [S–(L+M) and L–M]. The third bar corresponds to the regression on the L–M component alone. The last bar (light gray) shows the variance explained by the contrasts between hue similarity to red and blue. The red-versus-blue component explains more variance than does the L-M component and similar amounts of variance as the three biological components together

To test the biological component model, we then calculated regressions on the weights along the biological components to predict the overall sexual contrasts. The overall sexual contrasts are the sexual contrasts of Papuan and Polish observers together (average of the black and green curves in Fig. 4). Figure 5 illustrates the results for modeling the sexual contrasts in our study. The three dimensions of the second-stage mechanisms explained 70 % of the variance in the sexual contrasts of the favorite color choices, *R* ^2^ = .70, *F*(3, 8) = 6.3, *p* = .02. However, the two chromatic components used by Hurlbert and Ling (2007) yielded only marginally significant predictions, *R* ^2^ = .46, *F*(2, 9) = 3.9, *p* = .06. In regard to the single components, there was a significant positive correlation between the L–M component and the favorite color choices (41 %), *r* = .64, *p* = .03, but none for the S–(L+M), *r* = .32, *p* = .31, and the luminance component, *r* = .03, *p* = .94.

However, the sexual differences in Fig. 4 seem to contrast red and blue colors: The minimum of the two curves together (overall sexual contrasts; cf. Fig. S1) is around blue, implying that men like blue much more than do women; the maximum is at red, which means that women like red much more than men. Red-and blue do not coincide with the second-stage mechanisms. Thus, we determined the blue-versus-red contrast of each stimulus color as an alternative to the biological component model.

We calculated the relative hue distance of a given color to blue and red as the distance in degree azimuth to blue divided by the distance in degree azimuth between blue and red (for the respective arc of the hue circle). This relative hue similarity provides a perceptual component that varies between a red and a blue pole. This single component explained 72 % of the variance in the overall sexual contrasts, *r* = .85, *p* < .001, which indicates that the sexual contrasts depend on the perceptual similarity of each color to red and blue. The redder a color, the more it is liked by women and disliked by men, and vice versa for the similarity to blue.

## Discussion

Our results show that there are systematic differences in color preferences between the highly industrialized Polish and the nonindustrialized Papuan people (cf. Fig. 2). In contrast to the Polish observers, the Yali did not prefer blue, but red and yellow. The cross-cultural differences are in line with previous studies (e.g., Saito, 1996; Taylor et al., 2012) and may be explained by cultural or ecological factors, such as object–color associations (Palmer & Schloss, 2010b, 2010a).

Despite the pronounced cultural differences in overall color preferences, the sexual contrasts of favorite color choices were highly correlated in both cultures (*r* = .93). This high correlation shows that the difference in color preferences between women and men was almost the same in both cultures. These findings disagree with those of Taylor and colleagues (2012), who did not find any regularities of sexual differences across cultures. The question arises as to whether the regularities we found are an artifact of our simplified stimulus set and procedure.

First of all, if our approach yielded distorted color preferences, both the Polish and the Papuan color preferences should be affected. However, our results for the Polish observers were in line with those obtained in other industrialized societies. Our Polish observers preferred blue most, as do people in other industrialized societies (e.g., Palmer & Schloss, 2010b; Saito, 1996). Additionally, the correlations between the Polish preferences and the two biological components were similar to those Hurlbert and Ling (2007, p. R625) obtained for English and Chinese observers.

Only the fact that our Polish observers disliked orange-yellow most was apparently different from the results of previous studies. Observers in previous studies disliked greenish yellow (chartreuse) more than reddish yellow. This difference may be explained by the fact that reddish yellow hues become more and more aversive with decreasing lightness. When accounting for our bright white-point, our orange-yellow color was indeed darker (L* = 61.8) than in other studies (e.g., dark orange in Palmer & Schloss, 2010b; L* = 79.5).

More important, the cross-cultural differences in our study show that our stimulus colors did not simply yield unusually stable color preferences. Instead, the regularities across cultures are inherent to the differences between women and men. These sex differences were in line with the results in Hurlbert and Ling (2007), who found that sexual contrasts were mainly based on the fact that women preferred colors toward the reddish and men toward the greenish pole of the L–M component. Despite the differences in stimulus colors and preference measurements, our results agree with these observations.

Nevertheless, we doubt that the correlations with the second-stage mechanisms really reflect the biological origin of sex differences in color preferences. Our results indicate that these correlations are not specific to the two chromatic biological components used by Hurlbert and Ling (2007). In our study, the luminance component was necessary to explain the variation of sexual contrasts (70 % vs. 46 % of variance). Since our stimuli varied in lightness, the luminance component is needed to completely represent color similarity.

However, any linear transformation of the biological components, such as tristimulus values (XYZ) or the linearized RGB values of the monitor, yields exactly the same results. Moreover, the three dimensions of other color-spaces that completely represent color similarity explain the sexual contrasts in our study equally well, as shown by additional analyses for chromaticity coordinates (xyY, *r* ^2^ = 73 %), CIELUV (*r* ^2^ = 75 %), and CIELAB (*r* ^2^ = 70 %). Such correlations simply occur when sexual contrasts change gradually as a function of color similarity.

Moreover, Hurlbert and Ling (2007) claimed that the L–M component explained sexual contrasts particularly well. However, we found that a single perceptual component that directly represents hue similarity to red and blue may explain the sexual contrasts in our data better than any single biological component (cf. Fig. 5). In fact, this red-versus-blue component explained more of the variance in sexual contrasts (72 %) than did the L–M component (41 %; see above) and even slightly more than all three second-stage mechanisms together (70 %). Hence, the correlation of color preferences with the second-stage mechanisms does not necessarily imply that biological components are a better predictor of color preferences than any other index of color similarity. Overall, the biological component model does not contribute anything more to the explanation of the sexual pattern in color preferences than does any other color space that represents color similarity.

The observation that sex differences for favorite colors are modulated by the similarity to red and blue may be a particularity of the stimulus set and the task used in the present study. A larger, more exhaustive sample of stimulus colors might show that the relationship between color similarities and sex differences in color preferences is more complicated, and does not simply follow a contrast between two particular colors (Palmer & Schloss, 2010a). Nevertheless, the high correlation between the sex differences in Polish and Papuan observers supports the idea that sex differences in color preference follow the same pattern across cultures, as was claimed by Hurlbert and Ling (2007) for English and Chinese observers. Future studies using more complete stimulus samples and tasks than ours are necessary to determine the exact cross-cultural pattern of sex-differences across the whole color space.

In this regard, it is an open question why Taylor and colleagues (2012) did not find the cross-cultural pattern of sexual contrasts with the nonindustrialized Himba. One possible reason might be that the color rendering on a computer screen has biased the Himba preferences toward more saturated colors. The resulting effect of saturation might have covered the sex-specific differences in hue preference in their study. Another possibility is that the color preferences of the Himba are subject to determinants that are stronger than those that determine the cross-cultural sexual pattern we found with the Yali observers. In any case, the strong differences in color preferences across cultures highlight the fact that cultural and ecological factors generally modulate color preferences. Cultural and ecological factors may also interfere with the cross-cultural sexual pattern to different degrees depending on the cultures involved in a study.

## Conclusion

Overall, we found that the difference in color preferences between women and men transcends the cultural boundaries of even extremely different cultures. This is the case despite the fact that color preferences clearly differed between these cultures. Moreover, our findings indicate that the sex differences are not necessarily grounded in biological determinants of color vision, as has been claimed in previous studies. These results raise the question about what might shape these sex differences apart from biologically determinants. The distribution of colors in our natural environment or the social function of color signals are candidate factors that may influence color cognition through experience, while transcending cultural boundaries. It is a challenge for further research to test the generality of our results and to find out the origin of the sexual pattern across cultures.

## Electronic supplementary material

Below is the link to the electronic supplementary material.

ESM 1 (PDF 154 kb)
