# Supplementary material for: Sex differences in color preferences transcend extreme differences in culture and ecology
Source: Psychon Bull Rev. 2014 Feb 26;21(5):1195–201. doi: 10.3758/s13423-014-0591-8 (PMC4181517; doi:10.3758/s13423-014-0591-8)
Supplement: Supplementary file 1 — (PDF 154 kb) [file 13423_2014_591_MOESM1_ESM.pdf]

SUPPLEMENTAL MATERIAL

Sex differences in color preferences transcend extreme differences in culture and ecology

Piotr Sorokowski • Agnieszka Sorokowska • Christoph Witzel

Table of Content

S1. Exact Stimulus specifications 1
S2. Sex differences 2
S3. Least favorite choices 2
S3.1 Cultural differences 2
S3.2 Sex differences 2
S3.3 Biological components 3
S3.4 Discussion 3

S1. Exact Stimulus specifications

Table S1. Chromaticity coordinates of stimulus colors. White point corresponds to the illumination measured on a reflectance standard, background to the white background of the color wheel. Chromaticity coordinates are based on the CIE1931 color matching functions for a 2° standard observer. →

Color	ID	x	y	Y	L*	a*	b*
White point	WP	0.3289	0.3489	380	100	0	0
Background	BG	0.3285	0.3465	284	89.3	0.8	-1.4
Blue-purple	BP	0.2315	0.1528	13	21.8	26.8	-40.6
Blue	B	0.1886	0.1444	22	29.2	22.3	-55.6
Green-Blue	GB	0.1864	0.2371	43	40.0	-14.2	-37.1
Green	G	0.3589	0.5027	77	52.1	-25.8	38.5
Yellow-green	YG	0.4578	0.4793	178	74.0	1.7	73.2
Yellow	Y	0.4573	0.4736	188	75.7	3.1	72.4
Orange-yellow	OY	0.5061	0.4393	115	61.8	23.3	65.8
Orange	O	0.5219	0.4195	88	55.3	29.8	57.4
Red-orange	RO	0.5626	0.3744	58	46.2	45.0	46.1
Red	R	0.5831	0.3412	46	41.3	54.1	37.2
Red-purple	RP	0.4912	0.3073	32	34.8	41.7	8.8
Purple	P	0.3141	0.1890	24	30.2	40.7	-33.5

S2. Sex differences

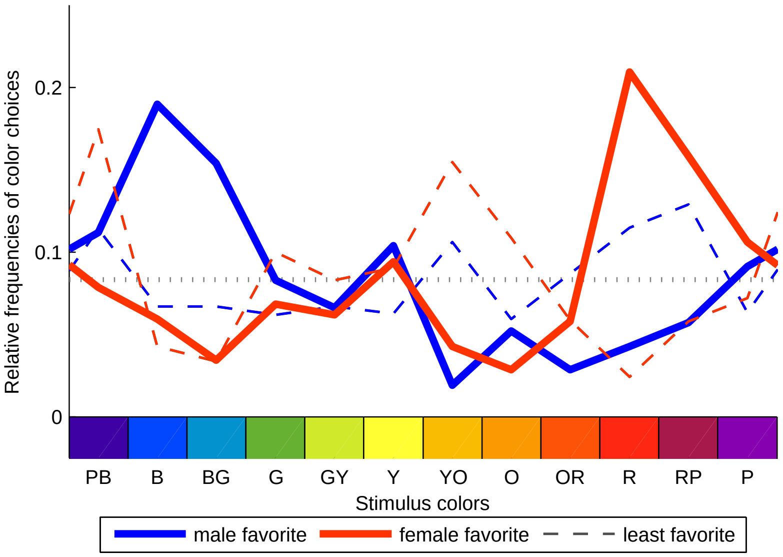

← Figure S1. Sex differences. Red curves correspond to female, blue curves to male color preferences, as averaged across cultures. Average relative frequencies of favorite (solid curves) and least favorite (dashed curves) color choices are shown along the y-axis. Other than this, format as in Fig. 2 of the main article. *Women (red curves) tended to prefer red, men (blue curves) blue.*

S3. Least favorite choices

S3.1 Cultural differences

Figure S2 presents the relative frequencies of least favorite color choices for the two cultures. As with favorite color choices, Polish and Papuan least favorite choices differed significantly in a chi-square test, $\chi^2(11,N=308)$=22.2, *p*=.02, and were not correlated, *r*=.02, *p*=.95. Moreover, the difference between Polish and Papuan least favorite color choices correlated highly between men and women, *r*=.73; *p*<.01, further supporting the observation that the cultural differences are consistent across sexes.

Colors that are frequently chosen as favorite colors should be selected less frequently as least favorite colors if both kinds of choices are based on the same preference judgment. Hence, the relative frequencies of favorite and least favorite colors should correlate negatively if they are consistent with each other. This was the case for the Polish observers, *r*=-.72, *p*<.01. Polish observers chose blue most rarely as the least favorite color (4.5%), and yellow-orange most often as the least favorite color (15.0%). This directly reflects the fact that Polish observers chose blue most frequently and yellow-orange most rarely as the favorite colors.

However, Yali least favorite choices were not correlated to the favorite choices, *r*=-.14, *p*=.66. Yali observers chose least frequently orange (2.8%) and most frequently blue-purple (20.4%) as least favorite colors. This contrasts the observation that they most frequently chose red and yellow, and most rarely yellow-orange as their favorite color.

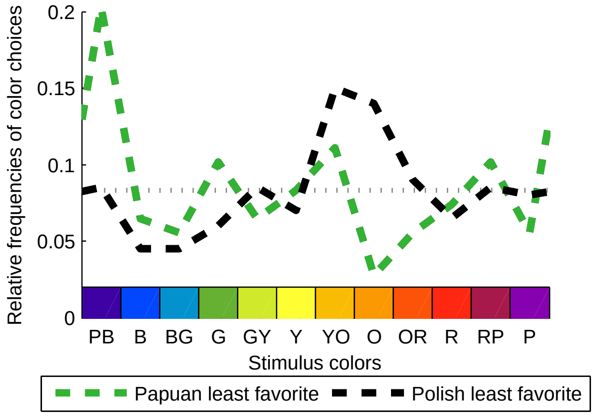

Figure S2. Cultural differences for least favorite colors. Thick dashed curves represent relative frequencies of least favorite color choices. Format as in Fig. 2. *As with the favorite color choices, least favorite choices differed between Polish and Papuan observers.*

S3.2 Sex differences

The thin, dashed curves in figure S1 show the least favorite choices of women and men for Polish and Papuan observers together. As for favorite color choices, women and men differed significantly in the relative frequencies of their least favorite color choices, $\chi^2(11,N=308)$=22.5, *p*=.02. The least favorite color choices also support the observation that women like and men dislike red since women chose red least (2.4%), and men chose red-purple and red most often as least favorite colors. The observation that men tend to like blue is reflected by a low frequency of men to select blue as a least favorite color. However, women do not chose blue most frequently as the least favorite color, but blue-purple (17.5%).

Figure S3 shows the least favorite color choices in each culture separately for women (panel a) and men (panel

b). For the favorite color choices (Fig. 3 of the main article), the difference between men and women was still significant when analyzing the Yali and the Polish group separately, and Polish and Papuan choices were correlated when analyzing women and men separately. These observations reflected systematic sex differences, and sex-specific patterns across cultures. However, for the least favorite colors sex differences were less systematic. The differences between women and men did not reach significance when analyzed separately in Papuan, $\chi^2(11,N=108)$=13.2, *p*=.28, and Polish observers, $\chi^2(11,N=200)$=13.2, *p*=.29. Moreover, neither the correlations between the least favorite color choices of Polish and Papuan women, *r*=.21, *p*=.53, nor those between Polish and Papuan men were significant, *r*=.23, *p* =.47.

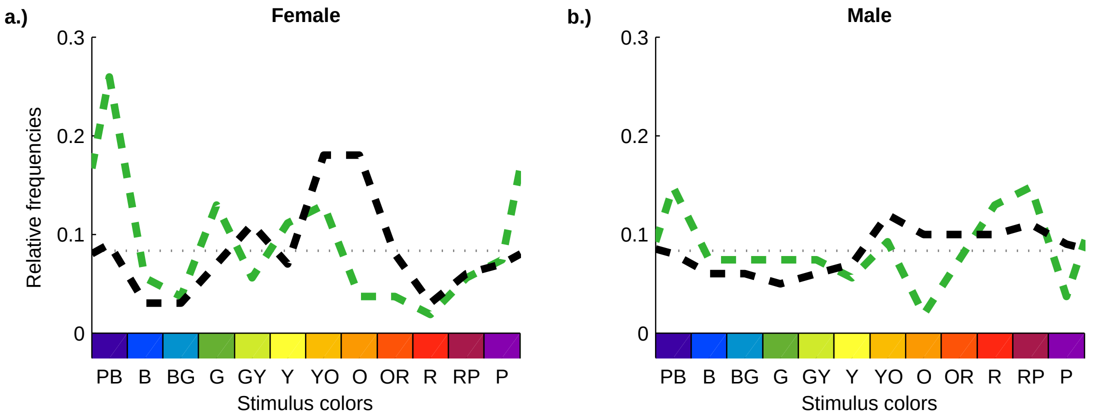

Figure S3. Sex-specific preferences across cultures for least favorite colors. Relative frequencies of Papuan (green curves) and Polish (black curves) least favorite color choices are shown separately for women (panel a) and men (panel b). Format as in Fig. 3.

Nevertheless, the main result of this study was also obtained with least favorite color choices. The main result consisted in a correlation between the sexual contrasts of Yali and Polish observers. As reported in the main article, this correlation was extremely high for favorite color choices (*r*=.93). Figure S4 shows the sexual contrasts of least favorite colors for Yali and Polish observers. The correlation between the sexual contrasts of Yali and Polish was lower for least favorite than for favorite colors, but still significant, *r*=.59; *p*=.045.

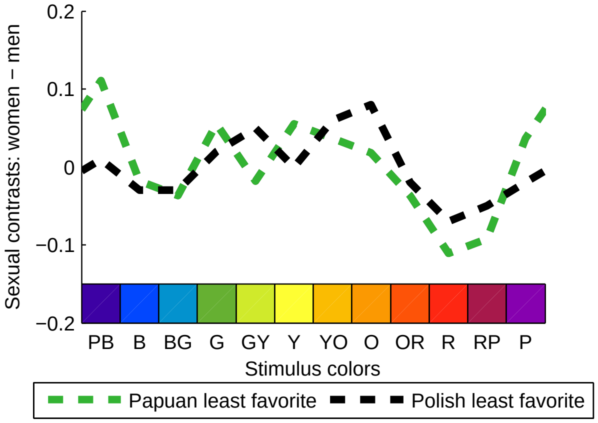

Figure S4. Sexual contrasts for least favorite colors. Thick dashed curves represent the differences between women's and men's relative frequencies of choosing the least favorite colors. Format as in Fig. 4. *As with the favorite color choices, sexual contrasts for least favorite colors were correlated between Polish and Papuan observers.*

S3.3 Biological components

The stimulus weights along the L-M component correlated positively with the least favorite choices of the Polish observers, *r*=.65, *p*=.02, reflecting the negative correlation found with their favorite color. The correlation between least favorite choices and the weights along the S-(L+M) component did not reach significance, *r*=-.45, *p*=.14. At the same time, the sign of this correlation (negative) was again inverse to the one with the favorite colors (positive).

The biological component model did not significantly predict the overall sexual contrasts of least favorite color choices, neither with two, R^2=23%, $F_{2,9}$=1.4, *p*=.31, nor with all three components, R^2=23%, $F_{3,8}$=.81, *p*=.52. The L-M component was the component that represented most of the variance of the sexual contrasts for our favorite color choices and in Hurlbert and Ling's (2007) study. For the least favorite choices in our study, this component did not correlate at all with the overall sexual contrasts, *r*=0. In the main article, we calculated the red-blue contrast to represent the variation of the sexual contrasts for favorite color choices as a function of hue similarity. If least favorite color choices were consistent with favorite color choices, the red-blue contrast should yield a negative correlation with the sexual contrasts of least favorite color choices. While the correlation coefficient was indeed negative, it was not significant, *r*=-.34, *p*=.29.

S3.4 Discussion

Least favorite color choices yielded overall fewer significant differences and correlations than favorite color choices. This is particularly true for the Yali least favorite color choices. In fact, further analyses showed

that least favorite color choices of either Papuan women or Papuan men did not yield any significant correlations that is neither with Papuan favorite color choices, nor with any kind of Polish color choices. In contrast, the least favorite choices of Polish observers were in line with their favorite color choices. This was even the case for women and men separately, r=-.62 and r = -.64; both p<.04. Moreover, the least favorite choices of Polish men correlated negatively with the favorite choices of Papuan men, r=-.63, p=.03. Hence, the least favorite choices of Polish men can predict the favorite color choices of both, Polish and Papuan men. Taken together, these results suggest that the Yali least favorite color choices (cf. dashed green curve in Fig. S2) were less consistent with the other measurements than other Polish and Papuan color choices (cf. other curves in Fig. S2).

One possible explanation for a lower consistency of Papuan least favorite choices might be that the concept of "least favorite color" was not always well communicated to the Yali, and caused misunderstandings in some of the Yali observers. In this case the favorite color choices would be more representative of cross-cultural patterns than the least favorite color choices.

However, despite the lower consistency and the fewer correlations, the least favorite color choices still reflected the main results found with the favorite color choices. Most importantly, there was still a correlation between the sexual contrasts in Polish and Papuan observers, supporting the idea of a cross-cultural pattern of sex differences.

Moreover, the least favorite color choices also disagree with the idea that biological components explain sex differences in color preferences apart from representing systematic variations as a function of color similarity. In fact, the biological component model fails to represent the sex differences of least favorite color choices. This may be explained by the fact that least favorite color choices do not vary gradually between a most and a least preferred colors. As can be seen in figure S4, most of the variation of the sex differences of least favorite colors (i.e. between purple-blue and orange-red) is irregular and there is no clear peak for a least favorite color.

Finally, even though the red-blue contrast did not significantly predict the sexual contrasts of least favorite colors either, it explains still more of their variance (12%) than the L-M component (0%). It is possible that the failure of the biological component model to predict the sexual contrast is due to potential misunderstandings of the concept of "least favorite colors" in Yali observers. Alternatively, it could be a particularity of least favorite color choices in general.

In any case, the results with the least favorite colors do not contradict the idea that the biological component model merely represents the relationship between color similarity and gradual changes in color preferences. Most importantly, the results with the least favorite colors further underpin the idea of a cross-cultural pattern of sex differences.
